# Characterization of Shallow Whole-Metagenome Shotgun Sequencing as a High-Accuracy and Low-Cost Method by Complicated Mock Microbiomes

**DOI:** 10.3389/fmicb.2021.678319

**Published:** 2021-07-30

**Authors:** Wenyi Xu, Tianda Chen, Yuwei Pei, Hao Guo, Zhuanyu Li, Yanan Yang, Fang Zhang, Jiaqi Yu, Xuesong Li, Yu Yang, Bowen Zhao, Chongming Wu

**Affiliations:** ^1^Beijing QuantiHealth Technology Co., Ltd., Beijing, China; ^2^Pharmacology and Toxicology Research Center, Institute of Medicinal Plant Development, Chinese Academy of Medical Sciences and Peking Union Medical College, Beijing, China; ^3^The Third Affiliated Hospital of Qiqihar Medical University, Qiqihar, China

**Keywords:** shallow whole-metagenome shotgun sequencing, 16S rRNA gene amplicon sequencing, metagenomics, mock microbiomes, consistency, accuracy

## Abstract

Characterization of the bacterial composition and functional repertoires of microbiome samples is the most common application of metagenomics. Although deep whole-metagenome shotgun sequencing (WMS) provides high taxonomic resolution, it is generally cost-prohibitive for large longitudinal investigations. Until now, 16S rRNA gene amplicon sequencing (16S) has been the most widely used approach and usually cooperates with WMS to achieve cost-efficiency. However, the accuracy of 16S results and its consistency with WMS data have not been fully elaborated, especially by complicated microbiomes with defined compositional information. Here, we constructed two complex artificial microbiomes, which comprised more than 60 human gut bacterial species with even or varied abundance. Utilizing real fecal samples and mock communities, we provided solid evidence demonstrating that 16S results were of poor consistency with WMS data, and its accuracy was not satisfactory. In contrast, shallow whole-metagenome shotgun sequencing (shallow WMS, S-WMS) with a sequencing depth of 1 Gb provided outputs that highly resembled WMS data at both genus and species levels and presented much higher accuracy taxonomic assignments and functional predictions than 16S, thereby representing a better and cost-efficient alternative to 16S for large-scale microbiome studies.

## Introduction

The booming metagenomics enables direct cell-free analysis of microbial communities from environmental habitats and greatly benefits a vast range of fields, including human health, drug development, agriculture, and ecology. Getting the taxonomic profiles of the microbiome is a major concern for metagenomic investigations, which is mainly achieved through two sequencing approaches, 16S rRNA gene amplicon sequencing (16S) and deep whole-metagenome shotgun sequencing (WMS) (Turnbaugh et al., 2009; Qin et al., 2010). WMS can provide taxa information down to species and even strain levels as well as analysis of functional potentials, and the high cost and massive data work leave it inaccessible to many researchers, particularly ones performing large longitudinal studies. In contrast, 16S sequencing represents a more convenient and cost-efficient method, but its accuracy almost cannot pass through the genus level. To complement each shortcoming, researchers compromise to perform 16S sequencing at the beginning of studies to gain primary clues for the subsequent WMS studies. Therefore, this strategy requires 16S results to be sufficiently accurate and highly consistent with the WMS datasheet. Otherwise, the whole project led by 16S sequencing would progress astray if the preliminary information derived from 16S was imprecise. Nevertheless, the accuracy and consistency of 16S readouts with WMS outputs have not been thoroughly investigated, mainly because there are few sophisticated artificial microbiomes with defined taxonomic information and similar complexity to the real microbial samples.

High-resolution and low-cost sequencing methods in the microbiome field are constantly in urgent need to facilitate massive research. Shallow WMS sequencing (S-WMS) is emerging as a candidate for large-scale microbiome studies. The novel sequencing has been explored to provide more shallow depth than the classical WMS, aiming to tackle the high cost of WMS while maintaining its benefits of deep resolution. It is documented that S-WMS is more effective to recover species-level polymorphisms and functional traits, and it exhibits less amplification bias than 16S sequencing (Jovel et al., 2016; Hillmann et al., 2018). Importantly, it requires much less materials and simplifies sample processing and DNA operations, which can primarily reduce the overall cost comparable with 16S. These advantages clearly mark S-WMS as a better alternative to 16S sequencing for the initial investigations regarding the vast microbiome. In light of these advantages, it is quite necessary to elaborate the consistency and accuracy of S-WMS results so that the promising method could be acknowledged and widely applied.

A well-defined mock microbiome, particularly with similar complexity to the real microbial samples, is essential for researchers to verify the consistency and accuracy of metagenomic sequencing methods. The rapid progress in culturomics has recently assisted in establishing artificial microbial communities to mimic real microbiome samples, making it feasible and reliable to systematically evaluate each metagenomic sequencing method (Greub, 2012; Diakite et al., 2020). To date, either commercial mock communities (e.g., ZymoBIOMICS Standards and ATCC Microbiome Standards) or a self-constructed artificial microbiome have been involved in several previous studies (Earl et al., 2018; Cichocki et al., 2020; Ducarmon et al., 2020; Han et al., 2020), whereas these known microbial communities only consisted of 4–23 species—far less sophisticated than the natural human microbiome. Hence, complex mock communities with more diverse species are still long-awaited to facilitate in-depth and precise investigations.

In this study, we construct sophisticated artificial microbiomes containing more than 60 gut species with varied abundance similar to the content in real human fecal samples and comprehensively evaluate the accuracy and consistency of 16S/S-WMS results with WMS data in both real fecal samples and the constructed artificial microbiome. Our findings demonstrate that 16S analysis is significantly inferior to S-WMS in terms of consistency with WMS, recall rate, and accuracy at the genus level. Moreover, S-WMS does not just provided high-resolution analysis down to the species level but also exhibits large coverage of functional predictions, thereby corroborating S-WMS as a potential alternative to 16S in further large-scale metagenomics surveys.

## Materials and Methods

### Fecal Sample Collection

The volunteers contributing fecal samples were recruited as part of research protocol number 2020LL-3 approved by the Third Affiliated Hospital of Qiqihar Medical University. The study was performed in accordance with the Helsinki Declaration. An informed consent was acquired from all volunteers recruited into the study. The fresh stool samples were collected from 69 subjects and stored at −80°C before use.

### Preparation of Mock Microbial Communities

The bacterial species in mock microbial communities were obtained from Quantibio Microbio Seed Bank (QuantiHealth Technology, Beijing, China^1^), and they were isolated from human fecal samples and cultured under standard laboratory conditions. The taxa of the bacteria were identified by matrix-assisted laser desorption ionization-time of flight mass spectrometer (MALDI-TOF MS) and genome sequencing.

### DNA Extraction

The microbial genomic DNA of human fecal samples and cultured bacteria were extracted by DNeasy PowerSoil kit (QIAGEN) according to the manufacturer’s instructions and then subjected to 1% agarose gel electrophoresis for evaluation. Concentration and purity of microbial DNA were determined with NanoDrop 2000 UV-vis spectrophotometer (Thermo Fisher Scientific) and Qubit 3.0 fluorometer (Thermo Fisher Scientific).

### 16S rRNA Gene Amplicon Sequencing and Data Processing

The hypervariable regions V3–V4 of the bacterial 16S rRNA gene were amplified by an ABI GeneAmp^®^ 9700 PCR thermocycler (Applied Biosystems) with primer pairs 338F (5′-ACTCCTACGGGAGGCAGCAG-3′) and 806R (5′-GGACTACHVGGGTWTCTAAT-3′). The PCR amplification of 16SrRNA gene was performed as follows: initial denaturation at 95°C for 3 min, followed by 27 cycles of denaturing at 95°C for 30 s, annealing at 55°C for 30 s, and extension at 72°C for 45 s, and single extension at 72°C for 10 min, and end at 4°C. The PCR mixtures contain 5× TransStart FastPfu buffer (TransGen Biotech) 4 μL, 2.5 mM dNTPs 2 μL, forward primer (5 μM) 0.8 μL, reverse primer (5 μM) 0.8 μL, TransStart FastPfu DNA Polymerase (TransGen Biotech) 0.4 μL, template DNA 10 ng, and finally ddH_2_O up to 20 μL. PCR reactions were performed in triplicate. The PCR product was extracted from 2% agarose gel and purified using the AxyPrep DNA Gel Extraction Kit (Axygen Biosciences) according to manufacturer’s instructions and quantified using Quantus^TM^ Fluorometer (Promega).

Purified amplicons were pooled in equimolar and paired-end sequenced (2 × 300) on an Illumina MiSeq platform (Illumina) according to the standard protocols by Majorbio Bio-Pharm Technology Co., Ltd. The raw reads were deposited into the NCBI Sequence Read Archive (SRA) database. The raw 16S rRNA gene sequencing reads were demultiplexed, quality-filtered by Trimmomatic, and merged by FLASH with the following criteria: (i) The 300 bp reads were truncated at any site receiving an average quality score of <20 over a 50 bp sliding window, and the truncated reads shorter than 50 bp were discarded, reads containing ambiguous characters were also discarded. (ii) Only overlapping sequences longer than 10 bp were assembled according to their overlapped sequence. The maximum mismatch ratio of the overlap region is 0.2. Reads that could not be assembled were discarded. (iii) Samples were distinguished according to the barcode and primers, and the sequence direction was adjusted, exact barcode matching, two nucleotide mismatch in primer matching. Operational taxonomic units (OTUs) with 97% similarity cutoff were clustered using UPARSE (version 7.1^2^), and chimeric sequences were identified and removed. The taxonomy of each OTU representative sequence was analyzed by RDP classifier^3^ against the 16S rRNA database (e.g., Silva SSU128) using a confidence threshold of 0.7.

### Shotgun Sequencing

Library preparation for shotgun sequencing was performed using the KAPA HyperPlus Library Preparation kit (KAPA Biosystems) for fragmentation of input DNA following the manufacturer’s instructions. The libraries were quantified by using KAPA Library Quantification Kits (KAPA Biosystems) following the manufacturer’s instructions. Libraries were constructed with an insert size of approximately 350 bp, followed by high-throughput sequencing to obtain paired-end reads with 150 bp in the forward and afterward directions.

Shotgun sequencing was performed on an Illumina NovaSeq 6000 System (Illumina). Cluster generation, template hybridization, isothermal amplification, linearization, blocking, denaturing, and hybridization of the sequencing primers were performed according to the workflow indicated by Illumina.

### Quality Control of Shotgun Sequencing Data

Low-quality reads were removed from the raw data by using MOCAT2 (Roat et al., 2016). Sequencing adapters were removed by using Cutadapt software (version v1.14,-m 30). Then, the SolexaQA package was used to remove the reads with a threshold of less than 20 or length of less than 30 bp. The reads that could be aligned with the human genome (*Homo sapiens*, UCSC hg19) were cleaned by using SOAP aligner software (v2.21, -M 4 -l 30 -v 10) (Li et al., 2009), and the rest of the reads were used for further analysis. The clean reads were assembled by SOAP *de novo* software (an iterative De-Bruijn Graph *De Novo* Assembler) using the parameters of -d 1,-M 3,-R,-u,-F to get the scaftigs of at least 500 bp.

Genes were predicted using MetaGeneMark. A non-redundant gene catalog was constructed with CD-HIT using the parameters of c 0.95–aS 0.9 (Fu et al., 2012). The clean reads were mapped onto the gene catalog with the length of at least 100 bp using BWA software to calculate the gene abundance.

### Taxonomic Profiling

Microbial community composition was analyzed using Metaphlan2 software. The query reads were mapped against the reference genomes in the RefSeq database (version 82) with a 97% identity threshold. The reads that mapped to a single reference genome were labeled with the NCBI taxonomic annotation. The reads that matched multiple reference genomes were indicated by the last common ancestor of each label according to the NCBI taxonomy.

### Functional Profiling

For 16S rRNA sequencing data, the metagenomes of the gut microbiome were input from 16S rRNA sequences with PICRUSt (Phylogenetic Investigation of Communities by Reconstruction of Unobserved States). This method predicts the gene family abundance from the phylogenetic information with an estimated accuracy of 0.8. The closed OTU table was used as the input for metagenome imputation and was first rarefied to an even sequencing depth prior to the PICRUSt analysis. Next, the resulting OTU table was normalized by 16S rRNA gene copy number. The gene content was predicted for each individual. Then, the predicted functional composition profiles were collapsed into level 3 of KEGG (Kyoto Encyclopedia of Genes and Genomes) database pathways. Pathways present in <10% of the samples were not included in the comparison analysis.

For shotgun sequencing data, functional profiling was acquired by KEGG Orthology group (KO) annotations for RefSeq-derived genes from directly observed exhaustive gapped alignments. To improve the KO profile accuracy for low-abundance genes, the KO profiles were separately predicted from reference genomes and the predicted profiles were used to augment the estimates of low-abundance KOs as previously reported (Jovel et al., 2016; Hillmann et al., 2018).

### Statistical Analysis

Data are indicated by means ±SEM R software (version 3.5.1) was used for the statistical analysis. The significance among groups was assessed by one-way ANOVA followed by Newman–Keuls *post hoc* tests. *p* < 0.05 was considered statistically significant.

## Results

### Shallow WMS Provided More Consistent Taxonomic Assignments With WMS Than 16S Analysis

**FIGURE 1:**
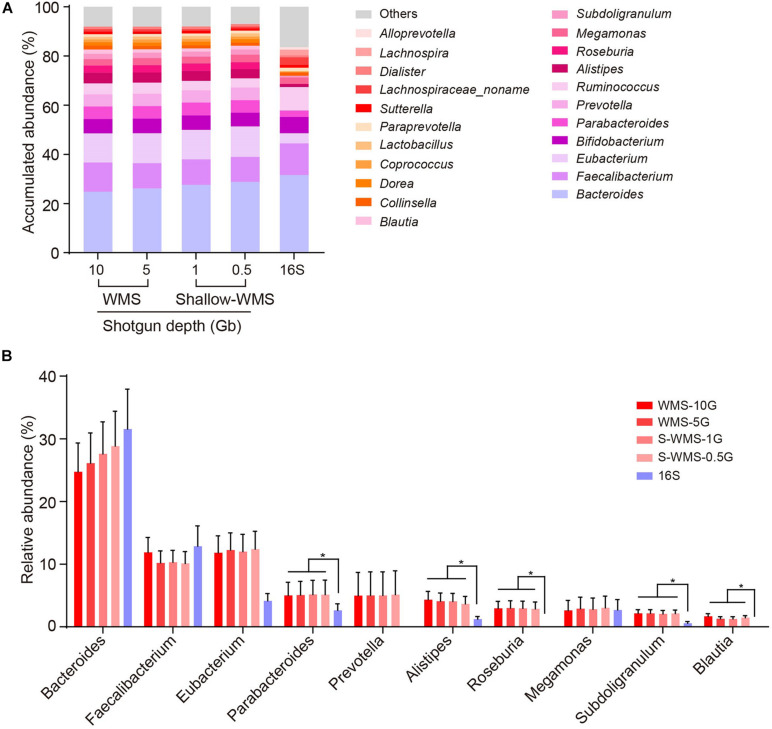
Comparison of genus profiling of human fecal samples by 16S rRNA gene amplicon sequencing (16S) or WMS sequencing with different depths. **(A)** Stacked bar plot of genera abundances. **(B)** The relative abundance of each key genus. The metagenomes of 10 human stool samples were sequenced by both 16S amplicon and WMS sequencing at 0.5, 1, 5, and 10 Gb depths, with the deep (10 Gb) shotgun sequencing as reference. *p < 0.05.

We adopted two cohorts of human fecal samples to evaluate the data consistency between shallow WMS/16S and WMS metagenomics. The first cohort, comprising 10 stool samples, was analyzed by 16S rRNA amplicon sequencing and WMS sequencing with different depths (10, 5, 1, and 0.5 Gb), respectively. Compared with the classical WMS data (10- or 5-Gb depth), both 16S and WMS with shallow depths of 1 and 0.5 Gb (referred to as S-WMS in the context) provided nearly identical results at the phylum, class, and order levels, but the family resolution of 16S sequencing was not very satisfactory (Supplementary Figure 1). At the genus level, the 16S-derived taxonomic profile varied from WMS in several genera, e.g., *Bacteroides* and *Eubacterium* (Figure 1A). Detailed statistics regarding dominant genera clearly display the disparate pattern of 16S from WMS analysis. According to 16S, abundant genera, including *Bacteroides* and *Faecalibaterium*, could be detected with no significant alterations, and the less abundant ones were markedly decreased (*Parabacteroides*, *Alistipes*, and *Subdoligranulum*) or even undetected (*Roseburia* and *Blautia*) (Figure 1B). In contrast, S-WMS showed nearly identical taxonomic information to WMS even at a depth of 0.5 Gb (Figures 1A,B). Although shallow sequencing depth (0.5 Gb) is conducive to the lower cost of a single sample, it requires many more samples to fulfill an entire chip (1 Tb), which may heavily delay the metagenomic process. Previous studies prove that approximately 1 Gb sequencing depth would provide sufficient metagenomic coverage (Jovel et al., 2016; Hillmann et al., 2018), so we selected 1 Gb as the optimal depth for S-WMS in the following studies.

**FIGURE 2:**
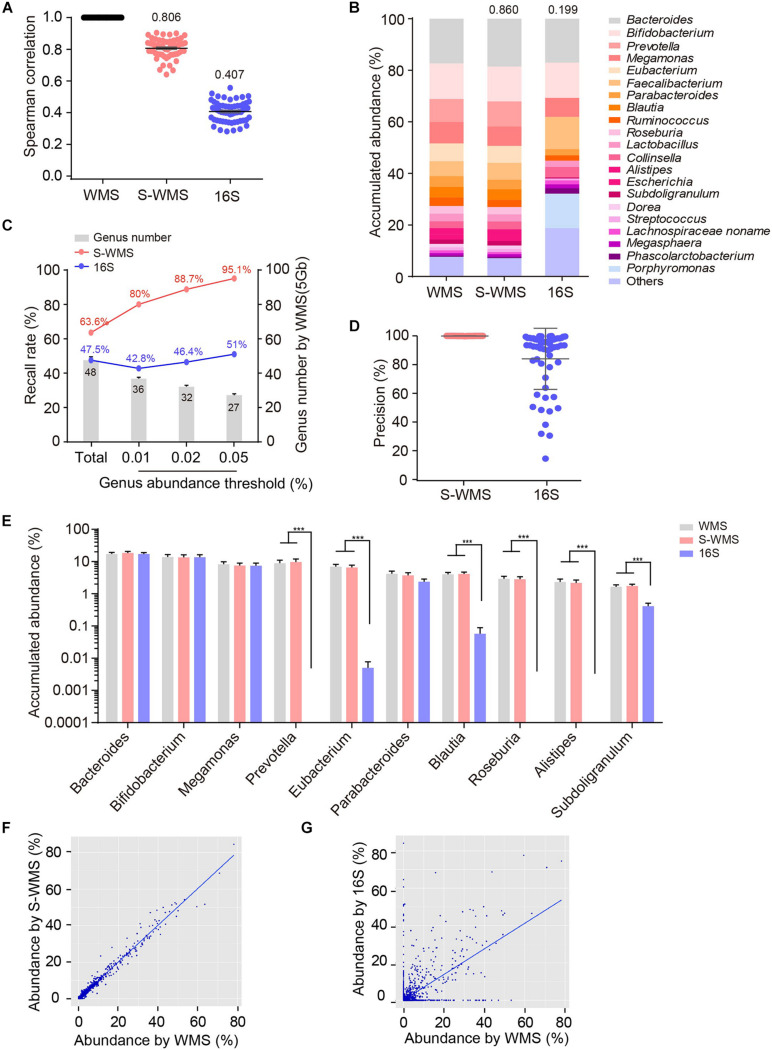
Comparison of genus profiling of human fecal samples by classical WMS, S-WMS, and 16S. **(A)** Spearman correlation coefficient between 16S/S-WMS and WMS results. The values of correlation coefficient from 59 samples are shown on top of the scatter plots. **(B)** Stacked bar plot of genera abundance. The values on top of the bar graph indicated the Spearman correlation coefficient between 16S/S-WMS and WMS results. **(C)** Genus recall rate increased with a higher genus abundance threshold. **(D)** Data precision to WMS results. Precision: the number of correctly matched reads with WMS/the total number of obtained reads. **(E)** Abundance quantification of key genera. **(F,G)** Scatterplot of relative abundances of genera determined by S-WMS or 16S versus classical WMS. The metagenomes of 59 human stool samples were sequencing by classical WMS (5 Gb depth), S-WMS (1 Gb depth), or 16S with the WMS as reference. ***p < 0.001.

To further confirm the consistency between S-WMS and WMS, we further took a larger cohort containing 59 human stool samples and found that S-WMS data was more highly associated with WMS than 16S-derived results (S-WMS versus 16S: 0.806 versus 0.407) (Figure 2A). When comparing the average taxonomic assignments among three sequencing approaches, the genera profile of 16S was obviously different from those of WMS and S-WMS (Figure 2B and Supplementary Figure 2) concomitant with the sharply declined similarity value 0.199 (Figure 2B). Furthermore, 16S recalled 47.5% of WMS-assigned genera, less than the high recall rate of S-WMS (63.6%). Significantly, the genus recall rate of S-WMS rose rapidly with a higher abundance threshold for counting genera, yet 16S showed little improvement (Figure 2C). Meanwhile, 99.9% of the S-WMS sequences correctly matched WMS reads, whereas 15.9% of the 16S data were not assigned by WMS (Figure 2D). For genus abundance quantification, 16S just presented similar levels of *Bacteroides*, *Bifidobacterium*, and *Megamonas* to classical WMS, accompanied by the significant reduce in *Prevetella*, *Eubacterium*, *Blautia*, *Roseburia*, *Alistipes*, and *Subdoligranulum* (Figure 2E). As expected, S-WMS displayed a highly consistent genera pattern with classical WMS regardless of each abundance (Figure 2E). Genera scatterplots of S-WMS/16S versus WMS further indicate that S-WMS results greatly resemble WMS data unlike the dispersed plot of 16S (Figures 2F,G). These results collectively demonstrate that there exists an apparent inconsistency between 16S and WMS data, and S-WMS sequencing is highly consistent with the classical WMS.

### Shallow WMS Revealed Accurate Taxonomic Results by Assessing Mock Microbial Communities

Given that the data inconsistency between 16S sequencing and WMS may be largely due to the inaccuracy of 16S or intrinsic biases of different sequencing approaches, we next constructed two types of artificial microbiome communities to evaluate the accuracy of different sequencing methods. The first artificial microbiome (MOCK1) contained 69 human gut bacterial species belonging to 33 genera of five phyla in which each species accounted for the same abundance. The second sample (MOCK2) comprised 62 human gut bacterial species belonging to 28 genera of five phyla, characterized by the varied abundance of each species, comparable to the average values of indicated species in human population (Supplementary Tables 1, 2).

**FIGURE 3:**
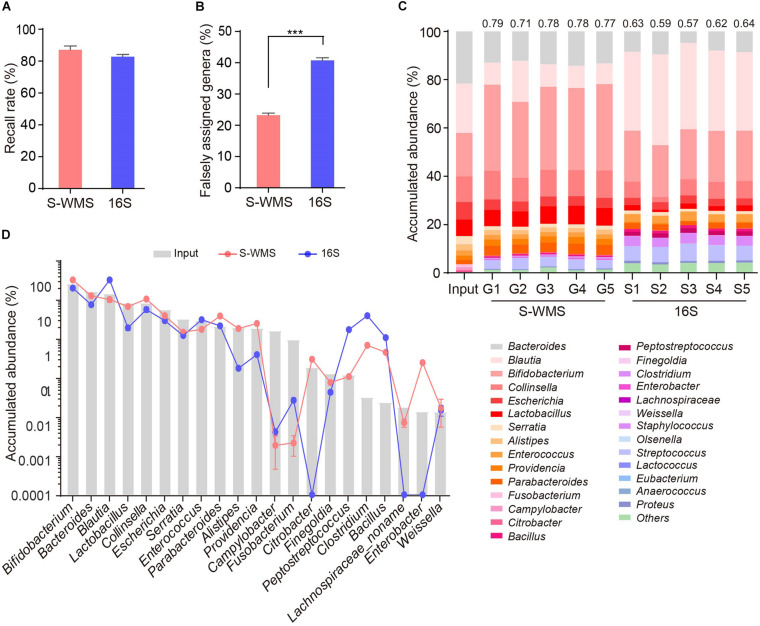
Genus composition of a high-complexity artificial microbial community (MOCK2) assessed by shallow WMS or 16S. **(A)** Genus recall rate. **(B)** Falsely assigned genus rate by 16S or S-WMS. **(C)** Stacked bar plot of genera abundances. The values on top of the bar graph indicate the Spearman correlation coefficient between S-WMS/16S data and the actual (Input) values. **(D)** The relative abundance of dominant genera determined by 16S or S-WMS. The gray bars indicate the actual (Input) value. Sixty-three gut bacterial species (belonging to 28 genera) were cultured under standard laboratory conditions. The artificial community was sequenced five times by each approach. ***p < 0.001.

For MOCK1 with lower complexity, both 16S and S-WMS recalled a high percentage of compositional genera (87.88 versus 95.96%) (Supplementary Figure 3A). However, about 37.78% of 16S-reported genera did not exist although only 12.03% of S-WMS-assigned genera were falsely recorded (Supplementary Table 3). Consequently, 96.46% of the S-WMS reads matched the real data, whereas up to 18.15% of 16S reads did not exist (Supplementary Figure 3B). Besides this, the genus abundance profile based on S-WMS was much closer to the actual one than 16S with an average similarity value of 0.87 (Supplementary Figure 3C). Metagenome analysis of MOCK2, whose composition largely mimics the real microbial community, also confirmed the high accuracy of S-WMS results. Although 16S recalled 82.86% of the compositional genera, slightly lower than the 87.14% of S-WMS (Figure 3A), 40.78% of 16S-assigned genera did not exist, significantly higher than the percentage of falsely assigned genera by S-WMS (28.64%) (Figure 3B). Consistent with observations using MOCK1, the S-WMS-assessed genus profile was also prominently closer to the actual pattern than 16S analysis (0.77 ± 0.03 versus 0.61 ± 0.03, *p* < 0.001) (Figure 3C). Of note, for the 19 genera with accumulated abundance of more than 0.01%, three genera, including *Citrobacter*, *Enterobacter*, and *Lachnospiraceae* were hardly detected by 16S sequencing, and S-WMS completely displayed all 19 genera (Figure 3D and Supplementary Table 4).

Therefore, according to assessments utilizing artificial microbiomes, our results reveal that S-WMS is more accurate for the genus assignments than 16S sequencing.

### Shallow WMS Displayed Precise Assignments Down to Species Level

**FIGURE 4:**
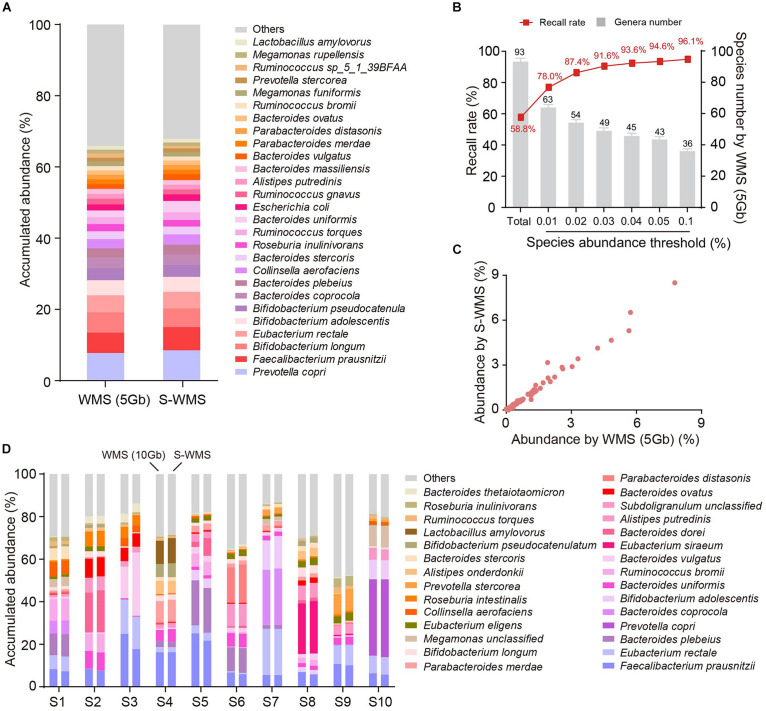
The species assignment provided by S-WMS and classical WMS. **(A)** Stacked bar plot of species abundances determined by classical (5 Gb depth) or S-WMS sequencing. **(B)** The recall rate of species types. The values on top of gray bars indicate the species numbers above each relative abundance threshold. The percentages on top of the curve nodes indicate the recall rate at each species abundance threshold. **(C)** Scatterplot of relative abundances of species determined by S-WMS versus classical WMS (5 Gb depth). **(D)** Stacked bar plot of species abundances in 10 typical fecal samples determined by WMS (10 Gb depth) or shallow (1 Gb depth) shotgun sequencing. The metagenomes of 59 human stool samples were sequenced.

Because the taxonomic resolution of 16S can rarely pass the species level, we next investigated whether shallow WMS also presented precise species information of microbiome. We observed that the species profile derived from S-WMS substantially resembled that of WMS with 5 Gb depth (Figure 4A). S-WMS recalled 58.5% of total species detected by WMS and the recall rate jumped to 91.6% for species with relative abundance >0.03% (Figure 4B). Moreover, the species abundance quantified by S-WMS was almost identical to that determined by WMS (Figure 4C), showing a high consistency between shallow and classical WMS regarding species assignments. As compared with a deeper WMS data (10 Gb), the consistency still remained, reflected by the similar estimation on abundance profiles at the species level (Figure 4D).

**FIGURE 5:**
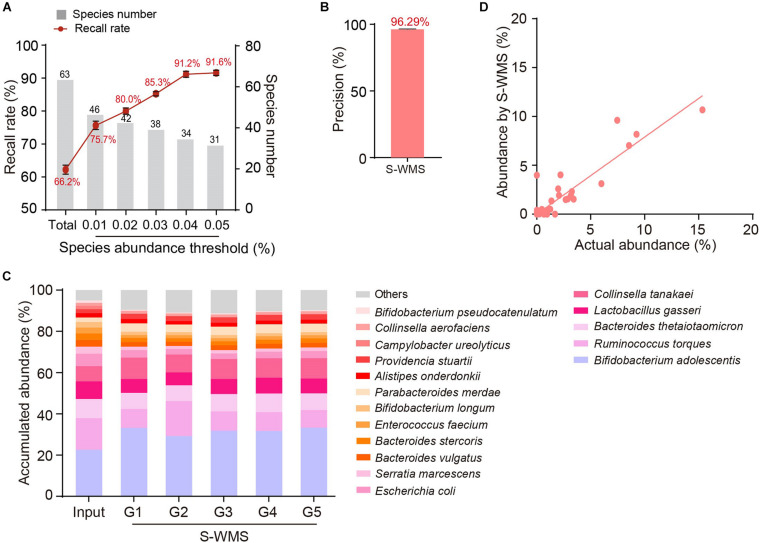
Species assignment of a high complexity artificial microbial community (MOCK2) using S-WMS. **(A)** The recall rate of species types. The values on top of gray bars indicate the species numbers above each relative abundance threshold. The percentages on top of the curve nodes indicate the recall rate at each species abundance threshold. **(B)** Data precision of S-WMS. The mean value is marked on top of the bar graph. **(C)** Stacked bar plot of species abundances determined by S-WMS versus actual (Input) values. **(D)** Scatterplot of relative abundances of species determined by S-WMS versus expected (Input) values.

We then introduced MOCK2 to verify the accuracy of S-WMS regarding species assignments. Although S-WMS recalled 66.2% of the total species in the MOCK2 community (Figure 5A), 96.29% of S-WMS reads were correctly assigned (Figure 5B). In particular, the recall rate sharply rose with the increase in species abundance. S-WMS could recall 91.2% of species with relative abundance >0.04% (Figure 5A). Moreover, S-WMS-deduced species profiles showed high similarity with the actual information, although with elevation in *Bifidobacterium adolescentis* (Figure 5C), and S-WMS provided credible abundance for most species (Figure 5D). Taken together, our results corroborate that S-WMS can provide credible species assignments, either WMS data or the artificial microbiome with defined taxonomic information as a reference.

### S-WMS Exhibited More Accurate Functional Prediction Than 16S Sequencing

**FIGURE 6:**
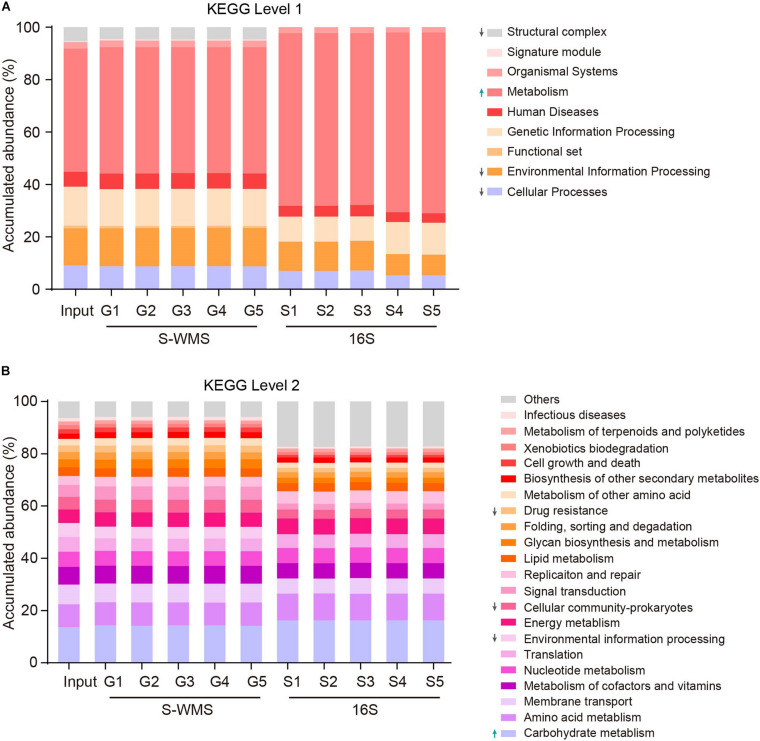
The functional repertoire prediction of MOCK2 by S-WMS and16S sequencing methods at KEGG levels 1 **(A)** and 2 **(B)** catalogs.

Prediction of the functional repertoire is another critical concern for microbiome analysis. Nowadays, the 16S data has been increasingly applied to predict the functional potential (Langille et al., 2013; Asshauer et al., 2015; Douglas et al., 2018), but its accuracy still needs further verification. Here, we utilized a complicated artificial microbiome whose overall function was well defined to evaluate the accuracy of 16S- and S-WMS-based functional prediction in terms of KEGG catalogs. The prediction by S-WMS was almost identical to the actual information (Input) at both KEGG levels 1 and 2 although 16S data displayed a varied prediction from the real profile at both levels 1 and 2 of KEGG catalogs (Figure 6). Specifically, the 16S-estimated functional proportions of cellular process, environmental information processing, and structural complex were much smaller, and metabolism function was larger than the real values at level 1 (Figure 6A). At level 2, the accumulated abundance of carbohydrate metabolism was increased and percentages of drug resistance, environmental information processing, and cellular community-prokaryotes were decreased as compared with the predicted profile of the MOCK2 community (Figure 6B). Besides this, at KEGG level 3, both 16S and S-WMS exhibited sufficiently reliable prediction of the microbiome (Supplementary Figure 4). Collectively, our results indicate that S-WMS is a more reliable method for the functional estimation of microbiome than 16S sequencing.

## Discussion

With the prosperity of metagenomics-based microbiome analysis, novel sequencing methods with low cost and reliable taxonomic and functional information are urgently needed, particularly for large longitudinal investigations. Recently, S-WMS has been emerging as a high-accuracy and low-cost candidate for large-scale microbiome studies (Jovel et al., 2016; Hillmann et al., 2018). However, the accuracy and consistency of S-WMS results with WMS data are currently not acknowledged, owing to the lack of solid evidence by well-defined mock microbiomes. In this study, we constructed artificial microbiomes containing more than 60 gut species with varied abundance similar to the content in real human fecal samples and comprehensively evaluated the accuracy and consistency of 16S/S-WMS results with WMS in terms of consistency, accuracy, and functional prediction. We provided conclusive evidence that S-WMS could present much more accurate taxonomic assignments and functional prediction with a low cost comparable to 16S sequencing. More importantly, S-WMS results are highly consistent with conventional WMS data, and their transition can be directly achieved on the same microbial samples without resampling.

It is a significant purpose for metagenome analysis to acquire knowledge about the taxonomy information of samples. Recall rate of taxon and quantification of abundance are two essential indicators for accurate assessments of microbiome. Although 16S remains a standard approach to characterize the taxonomic profile of various environmental samples, a growing body of evidence clearly shows the concomitant biases, such as uneven coverage of microbes’ diversity region by common 16S rRNA PCR primers (Hong et al., 2009), incorrect phylogenetic relationship within particular taxa (Dewhirst et al., 2005), and multiple 16S rRNA gene copy numbers in the genome (Kembel et al., 2012). Additionally, different DNA extraction protocols, especially the commercial kits, also have varied efficacy in extracting DNA from Gram-negative and -positive type strains (Sjöberg et al., 2020), which may result in considerable interventions to the following sequencing analysis. However, these biases are common for 16S rRNA and other marker gene-based sequencing methods, so it is difficult for researchers to thoroughly avoid them. In this work, we first carried out comparative analysis to evaluate the consistency of 16S/S-WMS with WMS. Intriguingly, 16S results displayed good consistency with WMS data at the phylum, class, order, and family levels but not down to the genus level. Only 47.52% of WMS-detected genera were recalled by 16S sequencing, and its recall rate did not increase much with the enrichment of specific genera. The taxonomic pattern of genera measured by 16S was also distinct from that determined by WMS. Several predominant genera with relative abundance >1.5% in human gut microbiota by WMS were even not detected by 16S, but some minimal genera, such as *Porphyromonas* was given a relative abundance value as high as 13.4%. Therefore, these remind us that close attention should be paid to this inconsistency when adopting a 16S-WMS combined strategy for microbiome characterization. On the contrary, S-WMS, as a promising candidate, could recall 63.66% of total genera assigned by classical WMS, and the recall rate sharply jumped to 88.80% for genera with abundance >0.02%. The genus profile reported by S-WMS also highly resembled the conventional WMS. Furthermore, S-WMS also provided a credible species-level taxonomy resolution of microbiota, especially species with relative abundance more than 0.01%.

Reliable accuracy estimation over a sequencing profiling largely depends on ideal artificial samples with specific taxonomic information that are sufficiently similar to real microbiome samples. Several studies evaluate the accuracy of 16S data, but their investigations were just based on data simulation (Escobar-Zepeda et al., 2018; Hillmann et al., 2018) or simple artificial microbiome samples, which comprise a small amount of bacterial species without abundance gradients (Jovel et al., 2016; Earl et al., 2018; Hillmann et al., 2018). In fact, more complex artificial microbiomes are crucial to assess the accuracy of various metagenome sequencing approaches. In our study, we established two types of artificial microbiomes, one consisting of 69 human gut bacterial species with equal abundance (MOCK1) and the other one comprising 62 species from 28 genera of five phyla (MOCK2). The relative abundances of compositional species ranged from 20.3539 to 0.0004% in MOCK2, corresponding to their authentic values in human population. To our knowledge, the MOCK2 microbial community is the most complicated microbiome and closest to the real human gut microbiota; thus, it could act as a benchmark for the systemic evaluation of various sequencing approaches. With the help of the sophisticated MOCK2 microbiome, we conclude that the taxonomic assignments of 16S sequencing was less precise, mainly based on the following evidence: first, about 40% of the total 16S-assigned genera did not exist in the sample; second, some dominant genera with relative abundance above 0.1% were not detected by 16S; third, some absent genera were reported by 16S with a large abundance (>1.50%). In contrast, S-WMS not only recalled a higher portion of compositional genera but also revealed more accurate taxon abundance. Eventually, the 16S-predicted genus profile was distinct from the real information, and the S-WMS-assigned genus composition was more similar to the input content.

In light of the defined compositions of MOCK2, we could obtain the authentic functional information of this microbial community. Although 16S data are increasingly used for functional prediction of microbiota (Langille et al., 2013; Asshauer et al., 2015; Xu et al., 2017; Douglas et al., 2018), its precision was not satisfactory after employing the complicated artificial microbiome into our investigations. We found that the functional profile deduced by 16S was markedly different from the actual information at both levels 1 and 2 of KEGG catalogs, and the S-WMS-provided functional description was quite similar to the actual data at both KEGG levels 1 and 2. Therefore, our findings did not just reminded researchers to take a cautious attitude toward 16S-predicted microbial functions, but it also proved S-WMS to be a reliable and cost-efficient method for functional estimation. Besides this, the application of the MOCK community indeed facilitated our understandings of the strengths and limitations of each sequencing approach, shedding novel light on the future studies.

## Conclusion

In summary, our work demonstrated that genus assignments and functional predictions by 16S sequencing were not accurate enough and not sufficiently consistent with WMS, according to assessments using both real fecal samples and a complicated artificial microbiome. In contrast, shallow WMS can provide a much more accurate description down to the species level, and highly resemble the classical WMS, representing it as a better alternative to 16S sequencing for large-scale studies.

## Data Availability Statement

The datasets presented in this study can be found in online repositories. The names of the repository/repositories and accession number(s) can be found in the article/Supplementary Material.

## Ethics Statement

The studies involving human participants were reviewed and approved by the Ethics Committee of the Third Affiliated Hospital of Qiqihar Medical University (2020LL-3). The patients/participants provided their written informed consent to participate in this study.

## Author Contributions

CW and BZ conceived the project and designed the experiments. TC and YP performed the experiments. WX assembled the figures and prepared and revised the manuscript. HG and CW performed the sequencing data analysis. YAY, FZ JY, YY, and XL assisted in manuscript preparation. ZL and BZ provided the gut bacteria samples for mock communities. All authors have read and approved the manuscript.

## Conflict of Interest

WX, TC, ZL, YP, HG, and BZ are employees of Beijing QuantiHealth Technology Co., Ltd. The remaining authors declare that the research was conducted in the absence of any commercial or financial relationships that could be construed as a potential conflict of interest.

## Publisher’s Note

All claims expressed in this article are solely those of the authors and do not necessarily represent those of their affiliated organizations, or those of the publisher, the editors and the reviewers. Any product that may be evaluated in this article, or claim that may be made by its manufacturer, is not guaranteed or endorsed by the publisher.

## Footnotes

1
https://microbe.quantibio.com/

2
http://drive5.com/uparse/

3
http://rdp.cme.msu.edu/

## Supplementary Material

Supplementary Figure 1 Comparison of taxon profiling of human fecal samples by 16S amplicon or WMS sequencing with different depths.

Supplementary Figure 2 The genus profile of 12 typical human stool samples assessed by 16S, S-WMS (1 Gb depth), and WMS (5 Gb depth).

Supplementary Figure 3 Genus composition of a low complexity artificial microbial community (MOCK1) assessed by S-WMS or 16S methods.

Supplementary Figure 4 The functional repertoire prediction of MOCK 2 at KEGG level 3 by S-WMS and 16S sequencing.

Supplementary Table 1 The species-level composition of MOCK1.

Supplementary Table 2 The species-level composition of MOCK2.

Supplementary Table 3 The genus profile of MOCK1 determined by 16S and S-WMS sequencing methods.

Supplementary Table 4 Genus quantification of MOCK2 by 16S and S-WMS methods.
