# Supplementary material for: Characterization of Shallow Whole-Metagenome Shotgun Sequencing as a High-Accuracy and Low-Cost Method by Complicated Mock Microbiomes
Source: Front Microbiol. 2021 Jul 30;12:678319. doi: 10.3389/fmicb.2021.678319 (PMC8361837; doi:10.3389/fmicb.2021.678319)
Supplement: Supplementary Figure 1 — Comparison of taxon profiling of human fecal samples by 16S amplicon or WMS sequencing with different depths. [file Data_Sheet_1.PDF]

Supplementary Material

Characterization of shallow whole-metagenome shotgun sequencing (S-WMS) as a high-accurate and low-cost method by complicated mock microbiomes

Wenyi Xu[2†], Tianda Chen[2†], Yuwei Pei[2], Hao Guo[2], Zhuanyu Li[2], Yanan Yang[1], Fang Zhang[1], Jiaqi Yu[1], Xuesong Li[3], Yu Yang[3], Bowen Zhao[2*], Chongming Wu[1*]

[1]Pharmacology and Toxicology Research Center, Institute of Medicinal Plant Development, Chinese Academy of Medical Sciences & Peking Union Medical College, Beijing, China.

[2]Beijing QuantiHealth Technology Co., Ltd., Beijing, China

[3]The Third Affiliated Hospital of Qiqihar Medical University, Qiqihar, China.

*** Correspondence:**

Chongming Wu, cmwu@implad.ac.cn;

Bowen Zhao, zhaobowen@quantibio.com.

[†]These authors contributed equally to this work.

This file includes：

Supplementary Figure 1 Comparison of taxon profiling of human fecal samples by 16S amplicon or whole-metagenome shotgun sequencing with different depths.

Supplementary Figure 2 The genus profile of 12 typical human stool samples assessed by 16S, S-WMS (1Gb-depth), and WMS (5Gb-depth).

Supplementary Figure 3 Genus composition of a low complexity artificial microbial community (MOCK1) assessed by S-WMS or 16S methods.

Supplementary Figure 4 The functional repertoire prediction of MOCK 2 at KEGG level 3 by S-WMS and 16S sequencing.

Supplementary Table 1 The species-level composition of MOCK1.

Supplementary Table 2 The species-level composition of MOCK2.

Supplementary Table 3 The genus profile of MOCK1 determined by 16S and S-WMS sequencing methods.

Supplementary Table 4 Genus quantification of MOCK2 by 16S and S-WMS methods.

Supplementary Figures

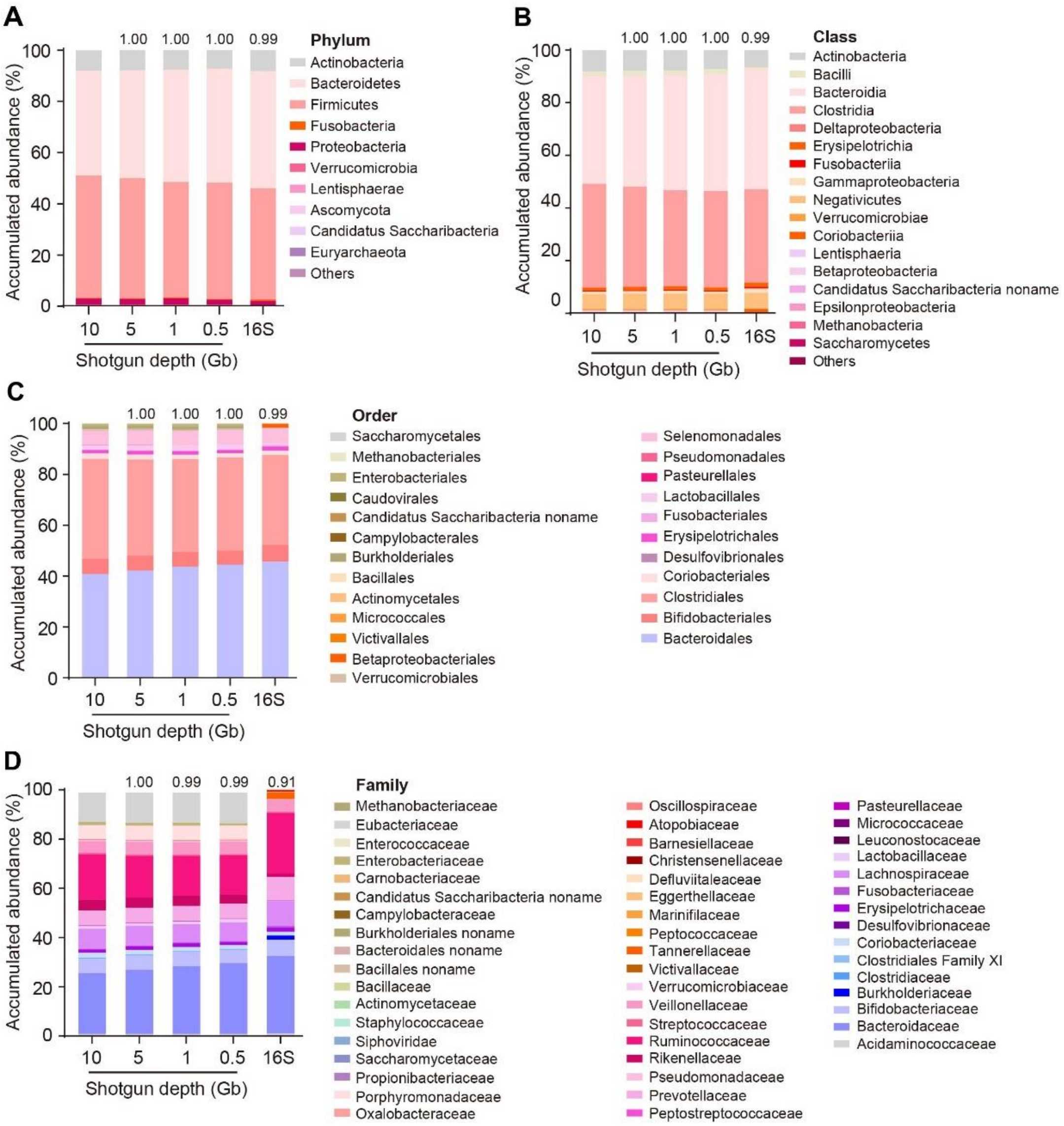

Supplementary Figure 1 Comparison of taxon profiling of human fecal samples by 16S amplicon or whole-metagenome shotgun sequencing with different depths. Taxonomic profiling at phylum **(A)**, class **(B)**, order **(C)**, and family **(D)** levels. The metagenomes of 10 human stool samples were sequenced by both 16S amplicon and whole-metagenome shotgun sequencing at 0.5Gb, 1Gb, 5Gb, 10Gb depths, with the deep (10Gb) shotgun sequencing as reference. The values on top of the bar graph indicated the Pearson correlation coefficient versus WMS (10Gb-depth) results.

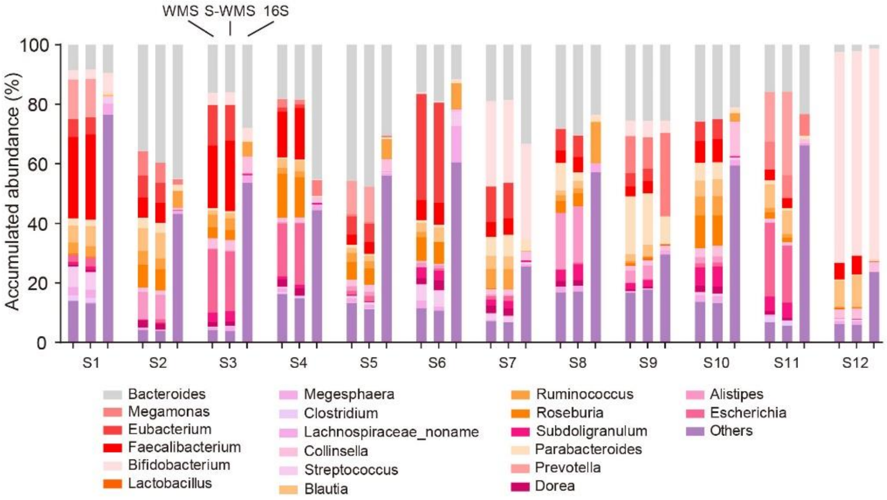

Supplementary Figure 2 The genus profile of 12 typical human stool samples assessed by 16S, S-WMS (1Gb-depth), and WMS (5Gb-depth).

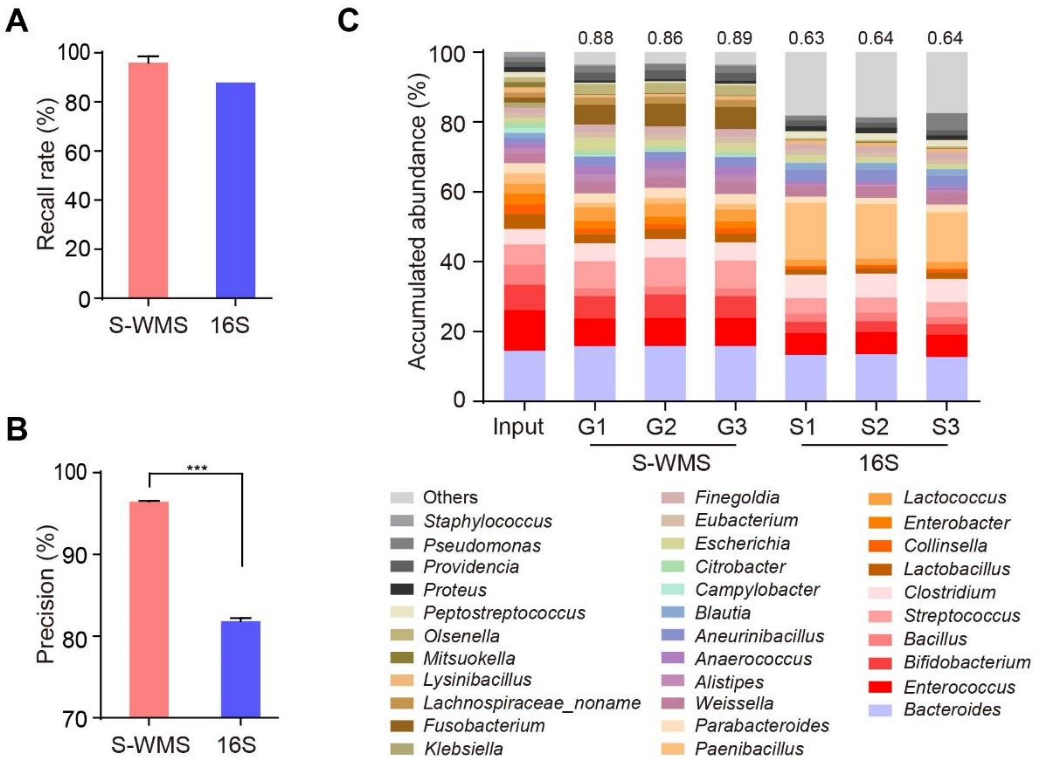

Supplementary Figure 3 Genus composition of a low complexity artificial microbial community (MOCK1) assessed by S-WMS or 16S methods. **(A)** Genus recall rate. **(B)** Data precision to classical WMS results. Precision = the number of correctly matched reads with WMS/total number of obtained reads. **(C)** Stacked bar plot of genera abundances. The values on top of the bar graph indicated the Spearman correlation coefficient between S-WMS/16S data and the expected (Input) values. Sixty-nine gut bacterial species (belonging to 33 genera) were cultured under standard laboratory conditions. The artificial community was sequenced three times by each approach. $^{***}p<0.001$.

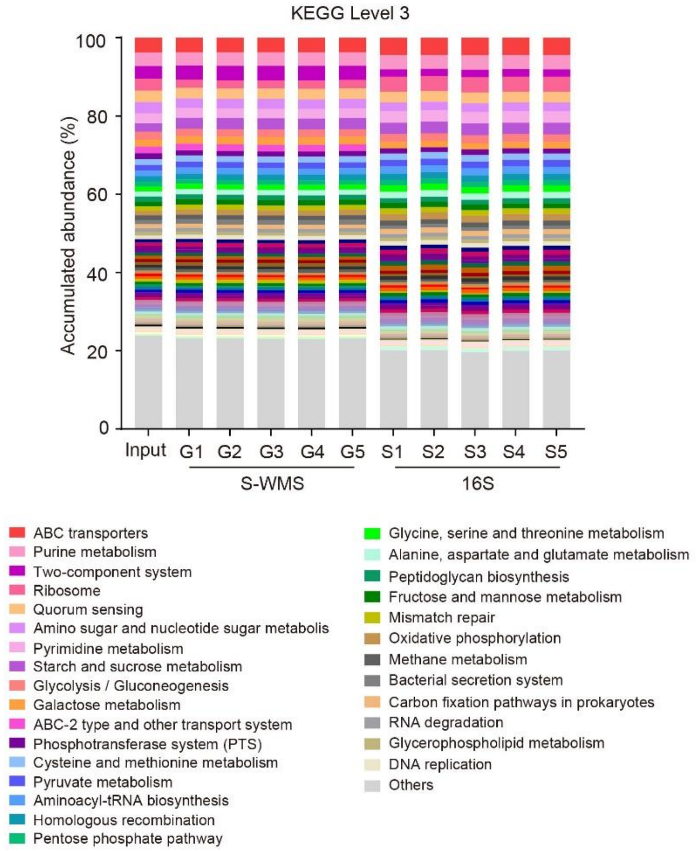

Supplementary Figure 4 The functional repertoire prediction of MOCK2 at KEGG level 3 by S-WMS and 16S sequencing.

Supplementary Tables

Table S1. The species-level composition of MOCK1.

Species	Actual Abundance(%)
Alistipes onderdonkii	0.0145
Anaerococcus vaginalis	0.0145
Aneurinibacillus aneurinilyticus	0.0145
Bacillus licheniformis	0.0145
Bacillus mojavensis	0.0145
Bacillus pumilus	0.0145
Bacillus subtilis	0.0145
Bacteroides faecis	0.0145
Bacteroides fluxus	0.0145
Bacteroides fragilis	0.0145
Bacteroides nordii	0.0145
Bacteroides ovatus	0.0145
Bacteroides salyersiae	0.0145
Bacteroides stercoris	0.0145
Bacteroides thetaiotaomicron	0.0145
Bacteroides vulgatus	0.0145
Bacteroides xylanisolvens	0.0145
Bifidobacterium adolescentis	0.0145
Bifidobacterium bifidum	0.0145
Bifidobacterium dentium	0.0145
Bifidobacterium longum	0.0145
Bifidobacterium pseudocatenulatum	0.0145
Campylobacter ureolyticus	0.0145
Citrobacter koseri	0.0145
Clostridium bifermentans	0.0145
Clostridium butyricum	0.0145
Clostridium perfringens	0.0145
Collinsella aerofaciens	0.0145
Collinsella tanakaei	0.0145
Enterobacter aerogenes	0.0145
Enterobacter cloacae	0.0145
Enterococcus avium	0.0145
Enterococcus casseliflavus	0.0145
Enterococcus cecorum	0.0145
Enterococcus dispar	0.0145
Enterococcus durans	0.0145
Enterococcus faecalis	0.0145
Enterococcus faecium	0.0145
Enterococcus raffinosus	0.0145
Escherichia coli	0.0145
Eubacterium limosum	0.0145
Finegoldia magna	0.0145
Fusobacterium mortiferum	0.0145
Klebsiella pneumoniae	0.0145
Lachnospiraceae bacterium 3 1 57FAA CT1	0.0145
Lactobacillus amylovorus	0.0145
Lactobacillus delbrueckii	0.0145
Lactobacillus sakei	0.0145
Lactococcus garvieae	0.0145
Lactococcus lactis	0.0145
Lysinibacillus sphaericus	0.0145
Mitsuokella multacida	0.0145
Olsenella uli	0.0145
Paenibacillus dendritiformis	0.0145
Paenibacillus sp PAMC 26794	0.0145
Parabacteroides distasonis	0.0145
Parabacteroides merdae	0.0145
Peptostreptococcus anaerobius	0.0145
Proteus penneri	0.0145
Providencia rettgeri	0.0145

Pseudomonas aeruginosa	0.0145
Ruminococcus torques	0.0145
Staphylococcus aureus	0.0145
Streptococcus anginosus	0.0145
Streptococcus infantarius	0.0145
Streptococcus infantis	0.0145
Streptococcus salivarius	0.0145
Weissella cibaria	0.0145
Weissella confusa	0.0145

Table S2. The species-level composition of MOCK2.

Species	Genome(Mb)	GC%	Actual Abundance (%)
Bifidobacterium adolescentis	2.2030	59.3	14.7214
Bifidobacterium bifidum	2.2034	62.7	0.5677
Bifidobacterium dentium	2.6358	58.5	0.1635
Bifidobacterium longum	2.3955	60	2.6739
Bifidobacterium pseudocatenulatum	2.2543	56.4	1.9023
Collinsella aerofaciens	2.2310	60	2.1900
Collinsella tanakaei	2.5099	60.35	8.4204
Olsenella uli	2.1532	64.7	0.0076
Bacteroides faecis	6.1465	42.45	0.0292
Bacteroides finegoldii	5.0046	42.9	0.3210
Bacteroides fluxus	4.3307	45.6	0.3402
Bacteroides nordii	5.5407	40.45	0.0350
Bacteroides ovatus	6.7168	41.9	3.0649
Bacteroides plebeius	4.0258	44.3	0.7794
Bacteroides stercoris	4.0801	46	3.3419
Bacteroides thetaiotaomicron	6.3729	42.9	8.7897
Bacteroides uniformis	4.9218	46.3	0.8400
Bacteroides vulgatus	5.0501	42.2	4.0942
Parabacteroides merdae	4.4751	45.3	2.1961
Alistipes onderdonkii	3.4023	58.4	3.1637
Bacillus cereus	5.7574	35	0.0244
Staphylococcus aureus	2.8369	32.7	0.0124
Enterococcus avium	4.4650	39	0.0004
Enterococcus casseliflavus	3.5554	42.6	0.0314
Enterococcus cecorum	2.3206	36.5	0.0095
Enterococcus faecalis	2.9686	37.4	0.1785
Enterococcus faecium	2.9207	37.823	1.8661
Lactobacillus amylovorus	2.0336	37.944	0.1274
Lactobacillus casei	3.0010	47.7	0.0252
Lactobacillus delbrueckii	1.8751	49.8	0.0283
Lactobacillus fermentum	2.0118	51.8	0.0182
Lactobacillus gasseri	1.9173	34.9	6.5480
Lactobacillus plantarum	3.2344	44.5	0.0038
Lactobacillus sakei	1.9926	41.065	0.0280
Weissella cibaria	2.4391	44.9	0.0067
Weissella confusa	2.2585	44.7	0.0073
Lactococcus garvieae	2.0437	38.5	0.0023
Lactococcus lactis	2.5114	35.1	0.0028
Streptococcus anginosus	1.9582	38.7	0.0012
Streptococcus cristatus	2.0731	42.4	0.0023
Streptococcus infantarius	1.9499	37.65	0.0024
Streptococcus infantis	1.8695	39.4	0.0013
Streptococcus macedonicus	2.1637	37.4	0.0022
Streptococcus mutans	1.9632	36.8	0.0012
Streptococcus sanguinis	2.3622	43.2	0.0010
Clostridium perfringens	3.4672	28.1	0.0135
Anaerococcus vaginalis	1.9980	29.6	0.0293
Finegoldia magna	1.9090	31.95	0.1348
Eubacterium limosum	4.3027	47.3	0.0441
[Ruminococcus] torques	3.0110	41.15	20.3539

Lachnospiraceae bacterium 3_1_57FAA_CT1	7.7128	46.8	0.0185
Peptostreptococcus anaerobius	2.0831	35.8	0.1236
Fusobacterium mortiferum	2.6813	29.1	0.9600
Fusobacterium ulcerans	3.5390	30.5	0.0198
Fusobacterium varium	3.3093	29.2	0.0212
Campylobacter ureolyticus	1.6657	29.1	1.6858
Citrobacter koseri	4.8240	53.8	0.1940
Enterobacter cloacae	4.9591	55.1	0.0142
Escherichia coli	5.1295	50.6	7.2924
Proteus penneri	3.7606	37.8	0.0012
Providencia stuartii	4.4921	41.4	1.9795
Serratia marcescens	5.1985	59.8	0.5402

Table S3. The genus profile of MOCK1 determined by 16S and S-WMS sequencing methods.

Genus	Actual abundance(%)	Estimated by S-WMS (1Gb)				Estimated by 16S			
		Falsely detected genera	ShotE1	ShotE2	ShotE3	Falsely detected genera	EMC1	EMC2	EMC3
Bifidobacterium	0.07		6.43	6.61	6.23		3.10	3.06	3.06
Collinsella	0.03		1.68	1.49	1.67		0.94	1.00	1.03
Olsenella	0.01		2.83	2.91	2.88		0.43	0.48	0.50
Bacteroides	0.14		15.74	15.86	15.87		13.31	13.48	12.68
Parabacteroides	0.03		2.75	2.82	2.77		1.85	1.71	2.16
Alistipes	0.01		2.04	2.07	2.08		0.35	0.32	0.50
Bacillus	0.06		2.23	2.28	2.19		2.49	2.42	2.01
Lysinibacillus	0.01		0.79	0.81	1.06		1.01	1.05	1.01
Aneurinibacillus	0.01		2.68	2.50	2.77		3.34	3.34	3.12
Paenibacillus	0.03		1.25	1.63	1.63		16.12	15.66	14.31
Staphylococcus	0.01		0.35		0.34		0.01	0.00	0.00
Enterococcus	0.12		7.93	8.08	8.03		6.26	6.38	6.38
Lactobacillus	0.04		2.56	2.78	2.51		1.51	1.54	1.84
Weissella	0.03		3.29	3.36	3.40		3.13	3.35	3.47
Lactococcus	0.03		3.93	3.78	3.48		1.88	1.83	1.92
Streptococcus	0.06		7.72	8.29	7.95		4.36	4.35	4.23
Clostridium	0.04		5.22	5.35	5.18		6.74	6.81	6.69
Anaerococcus	0.01		2.40	2.42	2.19		0.91	1.01	1.17
Finegoldia	0.01		2.23	2.33	2.27		1.77	1.73	2.02
Eubacterium	0.01		1.52	1.66	1.63		1.35	1.37	1.38
Blautia	0.01		0.24		0.23		1.95	1.92	1.92
Lachnospiraceae_	0.01		2.00	2.00	2.07				
Peptostreptococ	0.01		0.41	0.41	0.43		1.92	1.96	1.82
Mitsuokella	0.01		0.27	0.28	0.26		0.27	0.26	0.19
Fusobacterium	0.01				0.00		0.01	0.00	0.01
Campylobacter	0.01		0.30	0.30	0.33		0.06	0.06	0.06
Citrobacter	0.01		1.19	1.05	0.93				
Enterobacter	0.03		2.09	2.08	1.83				
Escherichia	0.01		3.80	1.96	2.77		2.19	1.64	1.33
Klebsiella	0.01		5.66	6.44	6.27				
Proteus	0.01		0.74	0.74	0.85		1.62	1.59	1.38
Providencia	0.01		2.23	2.50	2.26		1.48	1.49	1.40
Pseudomonas	0.01		1.88	1.85	1.99		1.42	1.43	4.92
		Corynebacterium	0.04	0.01	0.04				
		Dorea	0.28	0.10					
		Eggerthella	0.04	0.01	0.05				
		Peptostreptococcaceae_n	3.28	3.23	3.44				
		Siphoviridae_noname	0.35		0.34	Ralstonia	0.02	0.01	0.03

						unclassified_f__Enterobacte	8.85	8.95	7.71
		Total detected genera	37.00	34.00	37.00	Veillonella	0.03		
		Falsely detected genera	5.00	4.00	4.00	Virgibacillus	0.75	0.84	0.75
		False report rate (%)	13.51	11.76	10.81	Ezakiella	0.00	0.00	0.00
						Herminiimonas	0.00	0.00	0.03
						Leifsonia		0.00	
						Oceanobacillus	1.66	1.81	1.76
						Paeniclostridium	0.18	0.19	0.20
						Paraclostridium	3.75	4.00	3.87
						Peptoniphilus	0.15	0.19	0.22
						Phascolarctobacterium	0.03	0.05	0.07
						Porphyromonas	0.00		
						Actinomyces	0.26	0.20	0.24
						Akkermansia	0.00	0.00	
						Brevibacillus	1.39	1.41	1.42
						Butyricimonas	0.00		
						Corynebacterium_1	0.06	0.06	0.07
						Curvibacter	0.00		0.02
						Eggerthella	0.02	0.00	0.04
						Eisenbergiella	1.03	1.02	1.05
						Total detected genera	49.00	46.00	45.00
						Falsely detected genera	20.00	17.00	16.00
						False report rate (%)	40.82	36.96	35.56

Table S4. Genus quantification of MOCK2 by 16S and S-WMS methods.

Genus	Actual abundance (%)	Estimated by S-WMS (1Gb)					Estimated by 16SS					Deviation from the actual value (%)									
		FP50	FP5A	FP5C	FP5N	FP5V	MF1	MF2	MF3	MF4	MF5	FP50	FP5A	FP5C	FP5N	FP5V	MF1	MF2	MF3	MF4	MF5
Bacteroides	21.635	12.925	12.190	13.624	14.326	13.278	8.554	9.622	4.820	7.970	8.620	-40.26	-43.66	-37.03	-33.79	-38.63	-60.46	-55.53	-77.72	-63.16	-60.16
Blautia	20.420	9.242	17.005	9.304	9.170	8.616	32.613	37.519	35.775	33.272	32.493	-54.74	-16.72	-54.44	-55.09	-57.81	59.71	83.74	75.20	62.94	59.13
Bifidobacterium	18.013	35.637	31.458	34.421	34.025	35.813	21.108	21.452	20.627	21.152	20.880	97.85	74.64	91.10	88.90	98.82	17.19	19.10	14.52	17.43	15.92
Collinsella	10.610	11.820	9.898	11.053	10.761	11.303	6.721	2.138	6.669	6.946	7.214	11.40	-6.71	4.17	1.42	6.53	-36.66	-79.85	-37.14	-34.54	-32.01
Escherichia	7.292	4.292	4.082	3.996	4.002	4.173	2.904	3.157	3.342	3.004	2.813	-41.14	-44.03	-45.21	-45.12	-42.77	-60.18	-56.70	-54.17	-58.81	-61.43
Lactobacillus	6.779	6.794	6.277	7.264	7.662	7.236	2.301	0.963	2.298	2.007	2.377	0.22	-7.40	7.15	13.02	6.75	-66.05	-85.80	-66.10	-70.39	-64.94
Serratia	3.408	1.605	1.427	1.540	1.478	1.676	1.316	1.434	1.046	1.346	1.181	-52.92	-58.14	-54.80	-56.63	-50.82	-61.37	-57.94	-69.32	-60.51	-65.34
Alistipes	2.597	2.076	1.772	1.883	1.841	2.018	0.216	0.187	0.133	0.168	0.206	-20.05	-31.75	-27.46	-29.09	-22.30	-91.68	-92.80	-94.88	-93.52	-92.07
Enterococcus	2.086	1.818	1.802	1.759	1.944	1.790	3.215	3.078	3.734	3.023	3.146	-12.84	-13.60	-15.67	-6.80	-14.20	54.14	47.58	79.02	44.94	50.83
Providencia	1.883	2.601	2.358	2.629	2.761	2.570	0.473	0.418	0.261	0.449	0.431	38.08	25.22	39.60	46.59	36.47	-74.87	-77.83	-86.12	-76.18	-77.12
Parabacteroides	1.752	4.077	3.672	4.011	4.295	4.109	2.407	2.591	1.541	2.350	2.368	132.69	109.60	128.94	145.10	134.51	37.35	47.90	-12.03	34.15	35.14
Fusobacterium	1.262	0.006	0.002	0.000	0.000	0.002	0.038	0.021	0.025	0.020	0.033	-99.49	-99.81	-100.00	-100.00	-99.84	-96.99	-98.34	-98.00	-98.43	-97.37
Campylobacter	1.172	0.000	0.002	0.000	0.007	0.000	0.007	0.003	0.002	0.003	0.005	-100.00	-99.83	-100.00	-99.37	-100.00	-99.41	-99.72	-99.82	-99.72	-99.55
Citrobacter	0.659	0.303	0.335	0.304	0.277	0.332						-54.08	-49.20	-53.81	-58.01	-49.55					
Bacillus	0.097	0.477	0.458	0.463	0.478	0.463	1.177	1.064	1.145	1.066	1.119	394.58	373.92	379.18	394.83	379.18	1118.69	1002.45	1085.95	1003.70	1058.60
Peptostreptococcus	0.069	0.116	0.109	0.083	0.136	0.113	1.667	1.798	1.984	1.696	1.787	68.99	58.48	20.33	97.36	64.40	2323.15	2513.34	2783.77	2364.43	2497.06
Finegoldia	0.068	0.069	0.067	0.079	0.092	0.082	0.054	0.034	0.046	0.056	0.030	1.14	-0.57	16.43	35.09	21.66	-20.93	-50.01	-31.52	-17.21	-56.21
Clostridium	0.051	0.622	0.680	0.718	0.775	0.735	4.148	3.801	4.453	4.029	4.064	1120.27	1233.78	1309.21	1421.72	1342.37	8042.01	7360.48	8640.80	7809.21	7876.96
Enterobacter	0.047	0.212	0.283	0.186	0.299	0.291						355.52	507.72	298.65	542.14	525.18					
Lachnospiraceae	0.043	0.010	0.010	0.001	0.005	0.010						-77.27	-77.73	-96.73	-88.48	-76.22					
Weissella	0.014	0.002	0.008	0.000	0.013	0.063	0.026	0.015	0.000	0.023	0.012	-88.73	-39.68	-100.00	-5.29	354.98	87.22	4.85	-100.00	66.82	-11.76
Staphylococcus	0.012																				
Olsenella	0.010	0.015	0.003	0.003	0.012	0.004	0.038	0.011	0.034	0.035	0.035	49.88	-67.11	-74.89	16.89	-56.65	278.76	12.49	236.19	245.17	247.78
Streptococcus	0.007	3.674	4.634	4.058	4.181	3.515	6.050	6.315	7.125	6.388	6.053	53468.46	67476.49	59077.86	60863.42	51153.14	88124.41	91983.77	103791.18	93051.62	88168.39
Lactococcus	0.006																				
Eubacterium	0.006	0.003	0.004	0.005	0.001	0.000	0.031	0.018	0.023	0.033	0.024	-42.32	-36.50	-22.87	-89.03	-100.00	416.95	194.87	285.56	448.37	306.10
Anaerococcus	0.002	0.009	0.005	0.000	0.002	0.006	0.007	0.000	0.002	0.002	0.003	392.53	143.65	-100.00	-7.26	209.67	262.10	-100.00	10.48	-13.58	82.86
Proteus	0.001	0.414	0.365	0.408	0.411	0.431	1.004	0.934	0.786	0.915	0.928	32167.10	28358.18	31662.25	31939.60	33496.20	78101.28	72641.80	61172.33	71218.30	72223.20
others	0.000	1.181	1.093	2.208	1.048	1.370	3.925	3.427	4.126	4.048	4.177										
